# miR-500a-5p regulates oxidative stress response genes in breast cancer and predicts cancer survival

**DOI:** 10.1038/s41598-017-16226-3

**Published:** 2017-11-21

**Authors:** Davide Degli Esposti, Vasily N. Aushev, Eunjee Lee, Marie-Pierre Cros, Jun Zhu, Zdenko Herceg, Jia Chen, Hector Hernandez-Vargas

**Affiliations:** 1 Epigenetics Group. International Agency for Research on Cancer (IARC), 150 Cours Albert-Thomas, 69008 Lyon, France; 2 Department of Environmental Medicine and Public Health, Icahn School of Medicine at Mount Sinai, New York, NY 10029 United States of America; 3 Department of Genetics and Genomic Sciences, Icahn Institute of Genomics and Multiscale Biology, New York, NY 10029 United States of America; 4 Department of Pediatrics, Icahn School of Medicine at Mount Sinai, New York, NY 10029 United States of America; 5 Department of Medicine, Hematology and Medical Oncology, Icahn School of Medicine at Mount Sinai, New York, NY 10029 United States of America; 6 Department of Oncological Sciences, Icahn School of Medicine at Mount Sinai, New York, NY 10029 United States of America

## Abstract

MicroRNAs (miRNAs) are small regulatory non-coding RNAs with a diversity of cellular functions, and are frequently dysregulated in cancer. Using a novel computational method (*ActMir*) that we recently developed, the “activity” of miRNA hsa-miR-500a was implicated in estrogen receptor (ER) positive breast cancer; however its targets and functional impact remain poorly understood. Here, we performed an extensive gene expression analysis in ER+ breast cancer cell lines, to reveal the targets of miR-500a-5p after experimental modulation of its levels. We found that among mRNAs targeted by miR-500a-5p there was enrichment in oxidative stress response genes. Moreover, *in vitro* exposure to oxidative stress using H_2_O_2_ induces miR-500a-5p overexpression and downregulation of the oxidative stress targets *TXNRD1* and *NFE2L2*. Finally, expression of several of the identified miR-500a-5p targets related to oxidative stress, including *TXNRD1*, was associated with ER+ breast cancer survival in multiple datasets. Overall, we identify miR-500a-5p as an oxidative stress response miRNA whose activity may define breast cancer progression and survival.

## Introduction

MicroRNAs (miRNAs) are small non-coding RNAs that regulate gene expression in physiological and pathological conditions by degradation of messenger RNAs (mRNAs) or translational inhibition^1,2^. The spatial and temporal balance between miRNAs and their targets is tightly controlled, and its dysregulation plays a critical role in cancer development and progression^3^. Despite their importance, knowledge of direct and indirect targets of miRNAs is still limited, partially due to the technical and bioinformatic complexity of analyzing multiple messenger RNAs (mRNAs) targeted by one single miRNA and the redundancy of miRNAs targeting the same mRNA. Adding to this complexity, the cellular context influences the activity of a specific miRNA and the pool of its functional targets.

Dysregulation of miRNAs has been extensively reported in breast cancer^4,5^, as well as their potential as prognostic and predictive biomarkers^6^. The conventional way of identifying key regulatory miRNA is through examination of miRNA-mRNA relationships based on expression levels; however, such association does not imply causal relationship and miRNA expression cannot be equated to functional “activity”. We recently developed a computational approach, *ActMiR*, for identifying active miRNAs and miRNA-mediated regulatory mechanisms. Leveraging the data from the Cancer Genome Atlas (TCGA) dataset, we identified let-7d and miR-18a to be key microRNAs and prognostic markers for estrogen receptor negative and human epidermal growth factor receptor 2 negative (ER−/HER2−) breast cancer^7^. These key miRNAs were functionally defined by the association between their activity and the expression levels of a number of genes, and their prognostic value for breast cancer survival was confirmed in an independent dataset. Using a similar approach, the “activity” of miR-500a was also implicated in ER+ breast cancer mortality^7^. Recently, miR-500a has been linked to gastric cancer development^8^. However, while the “activity” of a miRNA cannot be directly measured but only inferred by its target genes, a complete target screening is not available for miR-500a; such information would be useful for understanding the role of miR-500a in breast cancer progression and improving the design of breast cancer survival predictors.

Here, we aimed to identify functional targets of miR-500a in breast cancer cell lines as well as to assess the predictive value of its expression in different breast cancer cohorts. Our data points towards regulation of oxidative stress response as a major pathway by which miR-500a influences breast cancer progression.

## Results and Discussion

### miR-500a is variably expressed across human breast cell lines

**Figure 1:**
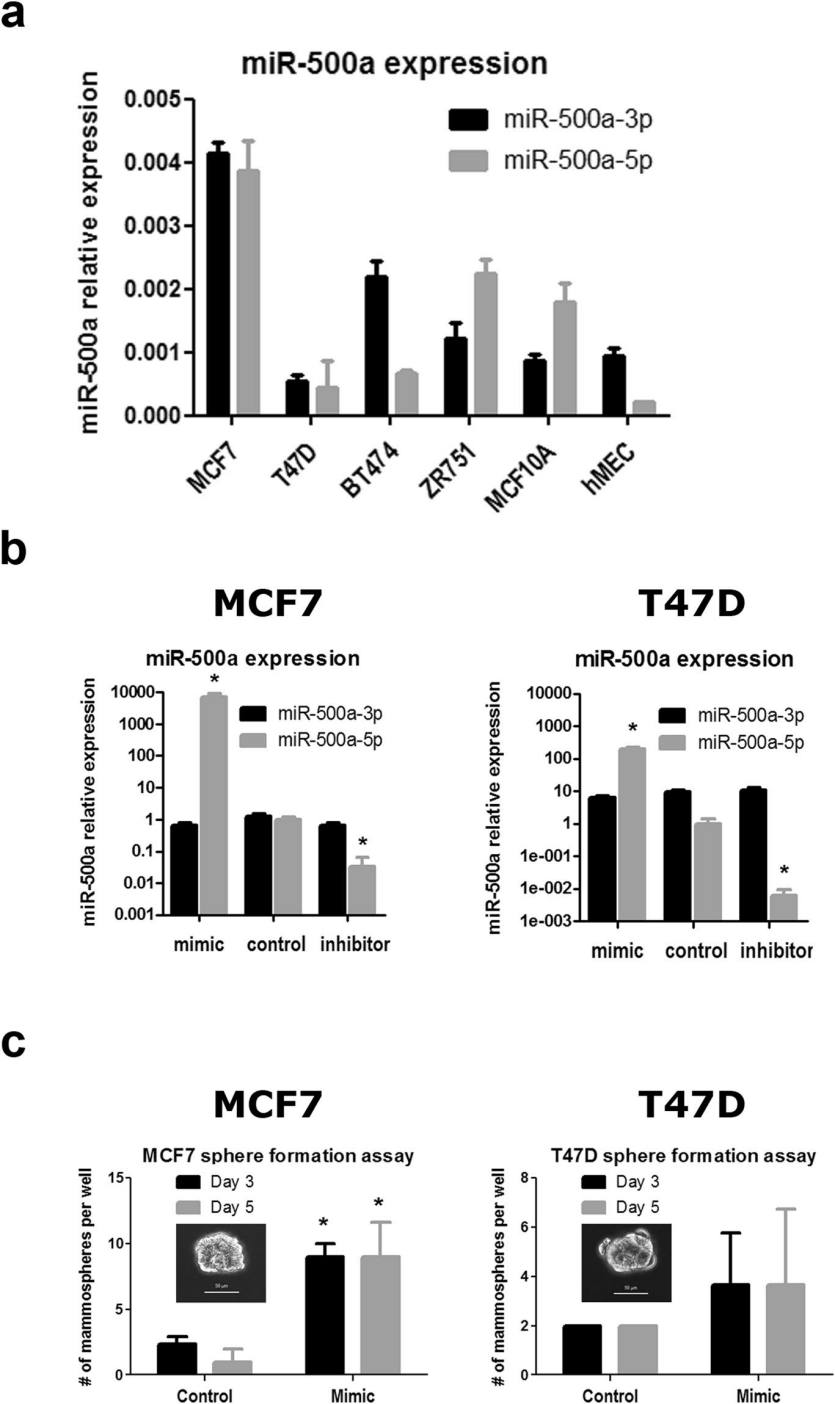
Modulation of miR-500a-5p expression in human breast cells. (**a**) Expression of guide (5p) and passenger (3p) strands of miR-500a was assessed by qRT-PCR in different breast cell lines. (**b**) Two breast cancer cell lines (MCF-7 and T47D) were selected for modulation of miR-500a-5p levels (gray barplots) after transfection of miR-500a-5p mimic and inhibitor. Expression of the passenger strand, miR-500a-3p (black barplots), was studied in parallel to define specificity. (**c**) Cells transfected with the same overexpression conditions shown in (**b**) were tested for sphere formation ability by growth in non-attachment plates. (*) indicates P value < 0.05 for the control vs. mimic comparison.

Similar to other miRNAs, the miR-500a precursor (pre-miR-500a) is processed into miR-500a-5p (MIMAT0004773, guide strand) and miR-500a-3p (MIMAT0002871, passenger strand). However, few data are currently available to determine *a priori* which is the functional strand. Therefore, we measured the expression of both miR-500a strands in four different ER+ breast cancer cell lines (i.e. MCF-7, T47D, BT474, and ZR751) and two non-cancerous immortalized breast cell lines (i.e. MCF10A and hMEC). MiR-500a-5p and miR-500a-3p expression levels are quite heterogeneous across the different cell lines, with both strands being detectable in all cell lines (Fig. 1a). MCF-7 cells display the highest level of expression of both strands, while T47D cells showed the lowest. Therefore, to cover a wider range of biologically relevant targets, we decided to use these two well-known breast cancer cell lines to modulate miR-500a expression and further study its role in ER+ cancer cells. In addition, a recent report showed that miR-500a-5p promoted gastric cancer cell tumorigenicity and was associated with malignant progression and poor survival^8^. Based on this, we decided to focus on the guide strand for the next analyses.

Our experimental strategy comprised both, suppression and overexpression of miR-500a-5p. Therefore, we expected an effect in both, miR500a-High MCF-7 cells and miR500a-Low T47D cells. We experimentally modulated miR-500a-5p levels by transfecting cells with a miR-500a-5p mimic (for overexpression) or a miR-500a-5p inhibitor (for silencing). As a negative control, cells were transfected with miRCURY LNA negative control, a miRNA designed to lack targets in the human genome (see Methods). MCF-7 cells transfected with the mimic miRNA showed increased levels of miR-500a-5p of several thousand-fold compared to the control, while cells transfected with the inhibitor miRNA showed decreased levels of miR-500a-5p of 30-fold (Fig. 1b). The efficiency of induction and suppression of miR-500a-5p was similar to what was previously reported for miR-30a overexpression and silencing in the same cell line^9^. Transfection with mimic and inhibitor in T47D induced a 100-fold overexpression and a 100-fold silencing (Fig. 1b). In both cell lines, modulation of miR-500a-5p expression did not affect the expression of the passenger strand miR-500a-3p (Fig. 1b), indicating a high specificity of the mimic and miR-500a-5p inhibitor.

Our examination of cell phenotype following transfection with miR-500a-5p mimic or inhibitor showed that modulation of miR-500a-5p expression did not influence cell morphology, cell death rate, or cell proliferation (data not shown). However, overexpression of miR-500a was associated with an increased ability to grow in non-attachment conditions (i.e. mammospheres) in MCF-7 cells (Fig. 1c). Such conditions have been associated with “stemness” properties of breast cancer cells. Although a similar trend was observed in T47D, this difference was not significant. This can be explained, at least partially, by lower miR-500a levels in this cell line at basal and overexpression conditions.

Taken together, these results show that miR-500a is variably expressed in human breast cell lines, and that its modulation does not affect cell death or cell proliferation at short time points. An effect in the ability to grow under non-attachment conditions is observed in MCF-7 cells.

### miR-500a-5p targets common transcripts in two breast cancer cell lines

Few studies have provided experimentally validated targets of miR-500a^8^, and no study is available in the context of breast cancer. Therefore, to identify the potential target genes of miR-500a in ER+ breast cancer cells, we adopted an unbiased approach by measuring whole transcriptome gene expression following modulation of miR-500a-5p levels by transfecting MCF-7 and T47D cells with miR-500a-5p mimic or miR-500a-5p inhibitor. After isolating RNA from each transfection condition (mimic, inhibitor or control miR) we interrogated gene expression using the Illumina HumanHT-12 v4 bead array, a technology that measures the expression of 31,000 annotated genes with more than 47,000 probes.

**Figure 2:**
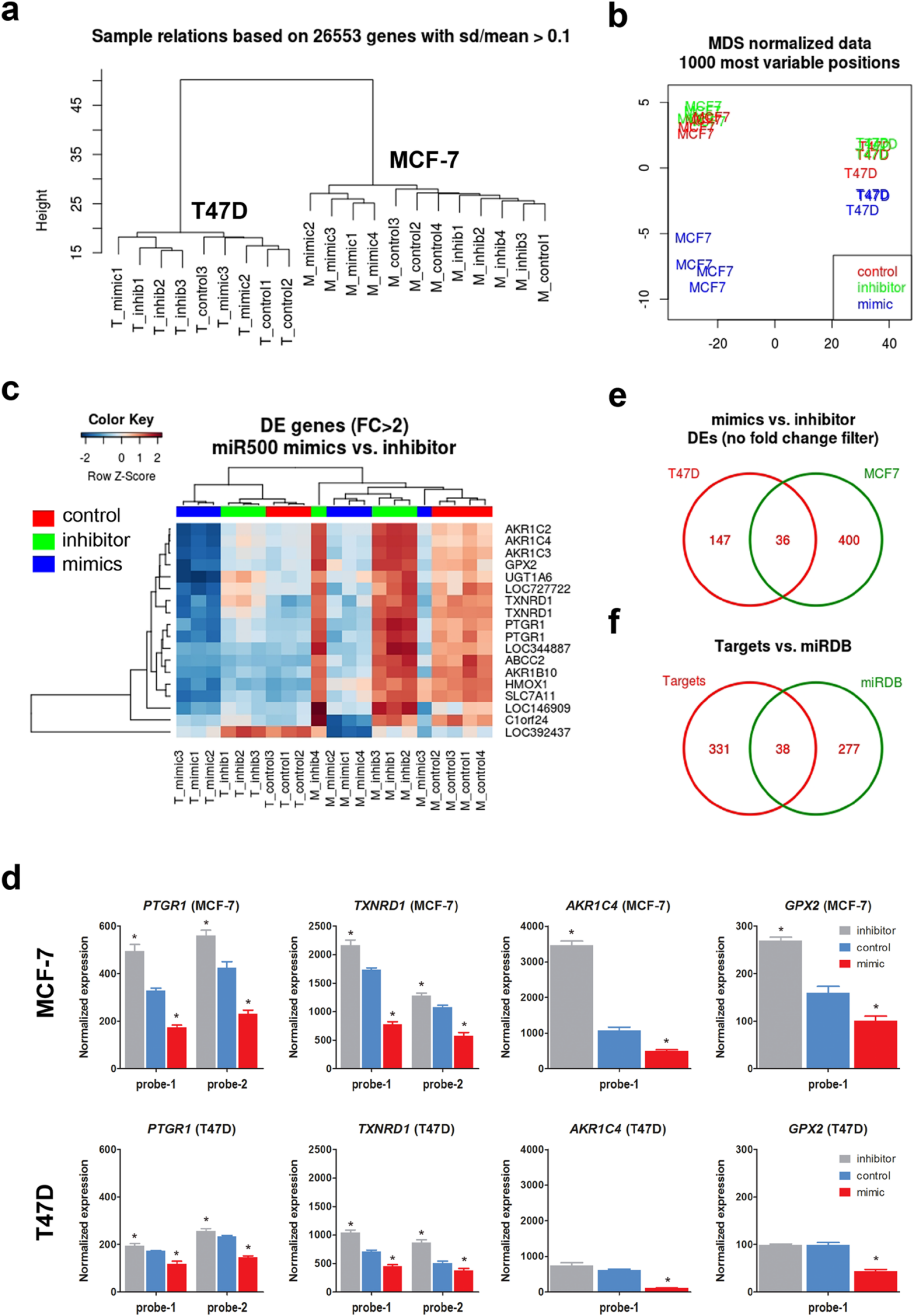
Genome-wide identification of miR-500a-5p target genes. Mir-500a-5p expression was modulated in MCF-7 and T47D cells by transfection of mimic and inhibitor, as shown in Fig. 1b. RNA obtained from each condition was used to assess gene expression using Illumina’s HT12 whole genome expression arrays. Unsupervised clustering (**a**), and multidimensional scaling (**b**) of gene expression data shows sample discrimination by cell type and treatment condition. Differentially expressed (DE) genes were obtained by comparing mimics vs. inhibitor in a linear regression model (see Methods). (**c**) Significant genes (FDR < 0.05) with a fold-change of at least two were used for a heatmap representation, where blue indicates low expression and red indicates high expression. T47D samples cluster on the left, while MCF-7 samples cluster on the right dendrogram. (**d**) A selection of DE genes displaying gradual expression across experimental conditions (i.e. control, mimic, or inhibitor) in the two cell lines. For *PTGR1* and *TXNRD1* two independent probes are shown. (**e**) Venn diagram representation illustrating the significant overlap between the targets identified in the two cells lines, when data is analyzed separately. (**f**) Venn diagram representation of the overlap between targets identified with our gene expression approach and those predicted with the miRDB web tool.

We next applied unsupervised clustering of the normalized expression data and found that expression profiles classify all samples according to the cell type (i.e. MCF-7 or T47D) (Fig. 2a). In addition, by plotting all samples according to the most variable probes (multidimensional scaling) we observed that within both cell lines there was a stronger effect of miRNA overexpression compared to miRNA inhibition (Figs 2b and S1). The divergent effect of mimics is more evident in MCF-7 cells, probably due to the higher efficiency of overexpression (several thousand-fold in MCF-7 vs. 100-fold in T47D).

To identify potential miR-500a-5p targets common to two ER+ breast cancer cell lines, we modeled the expression data as a function of cell type and experimental condition (see Methods). With this common design, we identified 413 differentially expressed transcripts between miR-500a-5p mimics and inhibitor (FDR-adjusted p value < 0.05) (Table S1). Most of these targets were downregulated after miR-500a-5p overexpression (n = 265), a fraction of which (n = 18) exhibited downregulation of more than two-fold (Fig. 2c and Table 1). Despite evident baseline differences between cell lines, the targets identified display a similar degree of downregulation relative to the non-treated conditions, in each case (Fig. 2c,d). Because of the global expression differences between both cell lines, we also performed separate analyses for each of them (Tables S2 and S3). Although more genes were differentially expressed in MCF-7 cells, there was a significant overlap between differentially expressed (DE) genes from both cell lines (eight times more than expected by chance, hypergeometric test p < 2.2 × 10^−22^) (Figs 2e and S1). The 36 common genes between both cell lines include those identified in the first analysis when using a fold-change threshold of at least two. For further discussion, we will refer only to the results of the combined analysis (modeling both cell types and all experimental conditions simultaneously) (Tables 1 and S1), as the robustness of the Bayesian approach applied here is known to benefit from including as many datasets as possible in the same analysis.

**Table 1:** miR-500a targets. List of differentially expressed probes (FDR < 0.05) after miR-500a modulation (miR-500a mimics vs. miR-500a inhibitor) in two breast cancer cell lines (i.e. MCF7 and T47D). Only transcripts with a fold change of at least 2 (|logFC| ≥ 1) are shown (n = 18 probes, corresponding to 16 genes). Transcripts belonging to oxidative stress/NRF2 pathways are underlined (n = 9). The last four columns show the P value and hazard ratios (HR) for the Kaplan Meier (KM) analysis of ER+ breast cancer survival using KMplotter and METABRIC datasets. KM p values below 0.05 are in bold.

Symbol	Probe_Id	logFC	FDR	KM plotter		METABRIC	
				P value	HR	P value	HR
*ABCC2*	ILMN_1676278	−1.09	1.1E-02	**2**.**10E-07**	**0**.**70 (0**.**61**–**0**.**80)**	0.115	1.33 (0.93–1.9)
*AKR1B10*	ILMN_1672148	−1.71	7.0E-04	0.069	0.88 (0.77–1.01)	**0**.**001**	**1**.**59 (1**.**19**–**2**.**12)**
*AKR1C2*	ILMN_2412336	−2.71	6.1E-07	**0**.**001**	**0**.**72 (0**.**59**–**0**.**87)**	**0**.**032**	**0**.**8 (0**.**66**–**0**.**98)**
*AKR1C3*	ILMN_1713124	−2.56	2.0E-08	0.120	0.90 (0.78–1.03)	**0**.**031**	**0**.**81 (0**.**66**–**0**.**98)**
*AKR1C4*	ILMN_1687757	−2.78	1.5E-07	**0**.**002**	**0**.**80 (0**.**69**–**0**.**92)**	**0**.**007**	**0**.**76 (0**.**61**–**0**.**93)**
*FAM129A*	ILMN_1667966	−1.02	2.0E-02	**2**.**20E-13**	**0**.**62 (0**.**55**–**0**.**71)**	**4**.**39E-03**	**0**.**72 (0**.**57**–**0**.**9)**
*GPX2*	ILMN_2133205	−1.32	6.3E-06	**6**.**00E-03**	**0**.**82 (0**.**71**–**0**.**94)**	**0**.**003**	**1**.**4 (1**.**12**–**1**.**75)**
*HMOX1*	ILMN_1800512	−1.13	6.0E-05	**0**.**048**	**1**.**14 (1**.**00**–**1**.**29)**	**0**.**004**	**1**.**5 (1**.**14**–**1**.**97)**
*KIF18B*	ILMN_1726682	−1.41	1.8E-03	**5**.**50E-11**	**1**.**53 (1**.**35**–**1**.**75)**	0.147	1.28 (0.92–1.79)
*LOC344887*	ILMN_3210914	−1.25	3.1E-03	**1**.**30E-09**	**0**.**56 (0**.**46**–**0**.**68)**	NA	NA
*FTLP2*	ILMN_3288717	−1.04	2.2E-06	NA	NA	NA	NA
*HGD*	ILMN_3239725	−1.10	8.9E-07	**0**.**003**	**1**.**23 (1**.**07**–**1**.**41)**	0.194	1.14 (0.94–1.38)
*PTGR1*	ILMN_1704531	−1.17	5.5E-06	**1**.**90E-06**	**0**.**63 (0**.**52**–**0**.**76)**	**1**.**30E-04**	**0**.**7 (0**.**6**–**0**.**8)**
	ILMN_2225537	−1.09	8.9E-07				
*SLC7A11*	ILMN_1655229	−1.40	2.1E-05	0.074	0.89 (0.78–1.01)	**0**.**007**	**1**.**37 (1**.**09**–**1**.**72)**
*TXNRD1*	ILMN_1717056	−1.36	2.0E-08	**<1E-16**	**1**.**72 (1**.**52**–**1**.**96)**	**1**.**12E-05**	**1**.**6 (1**.**3**–**1**.**9)**
	ILMN_2324421	−1.17	4.5E-06				
*UGT1A6*	ILMN_1752813	−1.06	3.7E-05	**0**.**001**	**0**.**73 (0**.**60**–**0**.**88)**	NA	NA

We compared our list of 413 experimental miR-500a-5p targets (corresponding to 369 gene symbols) to a list of 315 predicted targets available at miRDB^10^. This webtool uses NCBI RefSeq sequences for identification of 3′-UTR sequences^11^, and target prediction with the MirTarget algorithm^12^. 38 genes overlapped between the two lists (six times more than expected by chance, hypergeometric test p value < 2.15 × 10^−18^) (Fig. 2f). Seven of the identified targets also overlapped with the original target list used to determine miR-500a activity^7^. A similar overlap was found with other algorithms for miRNA target prediction (Fig. S2).

Finally, we performed an *in silico* validation of our gene expression approach. To this end, we used the MiRonTop mining tool^13^ to infer the most enriched miRNAs in our expression dataset after miR500a-5p modulation (see Methods). For this particular analysis, we used the complementarity of the seed sequence to define a gene as a miRNA target, either in the UTRs (Fig. S2, left panel) or the coding sequence (CDS) (Fig. S2, right panel). Interestingly, miR500a-5p is significantly enriched only in the CDS targets. Importantly, miR500a-5p is the top most significant miRNA when taking together UTR and CDS targets. A similar result is obtained when using TargetScan^14^ as the reference dataset to define miRNA targets. In that case, miR500a-5p displays the highest enrichment among all human miRNAs.

We therefore identified 369 genes directly or indirectly targeted by miR-500a-5p in two ER+ breast cancer cell lines. Although these genes significantly overlap with predicted targets, many of them are described here for the first time. Our *in silico* validation gives strong support to our data. Moreover, they uncover a possible role of miR500a-5p in binding coding sequences for gene expression regulation. There is evidence for a biological role of miRNAs in such context^15,16^. A possible functional role, such as in splicing, could be an interesting guide for future research.

### miR-500a-5p putative transcriptional targets are enriched in oxidative stress pathways

Because high miR-500a activity has recently been implicated as a key microRNA in ER+ breast cancer^7^, we next used the transcriptional targets described above in ER+ breast cancer cell lines to identify functional pathways that could explain such clinical observation. Gene set enrichment analyses showed that genes differentially down-regulated after miR-500a-5p overexpression were enriched in oxidative response pathways, and specifically in targets of NRF2 (Table 1 and Fig. S3). The transcription factor NRF2 [also known as NFE2L2, nuclear factor (erythroid-derived-2)-like 2] induces the transcription of several genes involved in the cellular antioxidant response, and is therefore considered as a major regulator of cell survival^17^. Interestingly, in our list of identified miR-500a-5p targets, the top most significant enrichments (all with FDR < 0.05) were found to be related to “Oxidative Stress Induced Gene Expression Via Nrf2” (BioCarta), “NRF2 pathway Homo sapiens” (WikiPathways), “NFE2L2 ChIP-Seq” (ChEA 2015), and “NFE2L2 CHEA” (ENCODE and ChEA Consensus TFs). Enrichment in oxidative stress genes was observed for all significant genes (FDR < 0.05), regardless of the fold-change filter (i.e. no filter, or two fold-change filter). Within the 18 transcripts (16 annotated genes) with more than two-fold change after miR-500a-5p overexpression, nine are known NRF2 targets (corresponding to seven annotated genes: *SLC7A11*, *TXNRD1*, *GPX2*, *PTGR1*, *HMOX1*, *ABCC2*, and *UGT1A6)* (Table 1). These results show that oxidative stress response genes are major targets of miR-500a-5p in ER+ breast cancer cell lines.

### miR-500a-5p activity is modulated under oxidative stress conditions

**Figure 3:**
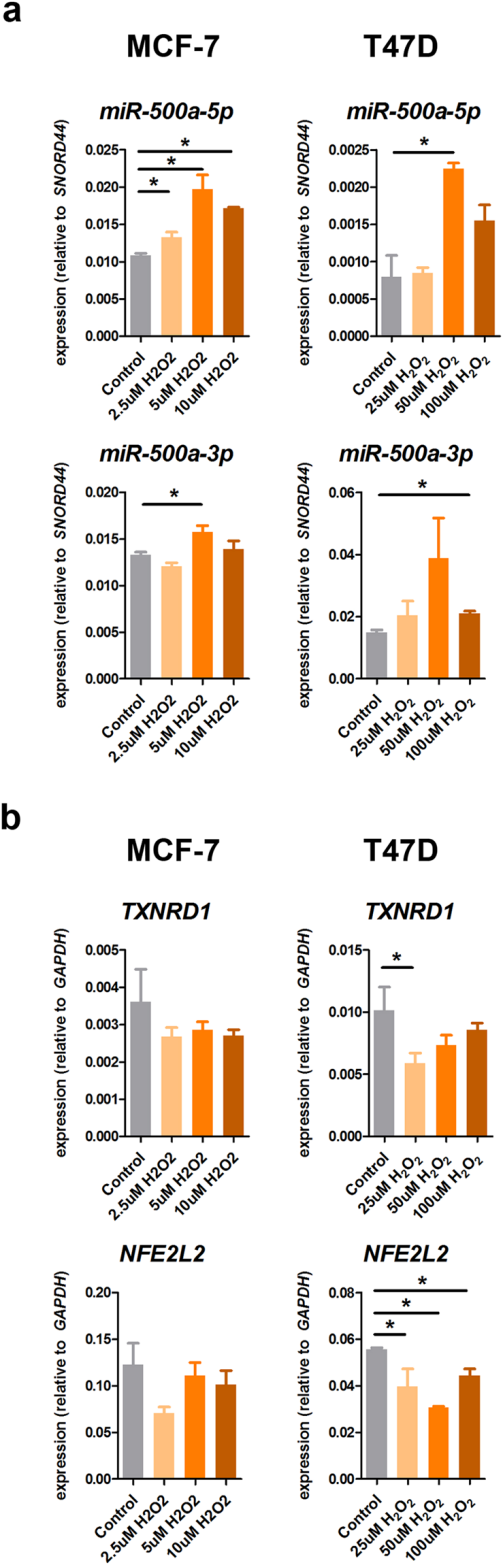
Role of miR-500a-5p in the response to oxidative stress. (**a**) MCF-7 and T47D cells were exposed to increasing concentrations of H_2_O_2_ within their respective sublethal range. RNA extracted under these conditions was reverse-transcribed and tested for expression of miR-500a-5p guide strand (upper panels), and miR-500a-3p passenger strand (bottom panels). (**b**) Two selected targets of miR-500a-5p (i.e. *TXNRD1* and *NFEL2L*) were studied under the same experimental conditions. (*) indicates P value < 0.05.

We next aimed to characterize a functional effect of miR-500a in modulation of oxidative stress response. To this end, we exposed T47D and MCF-7 cells to different concentrations of the reactive oxygen species (ROS) hydrogen peroxide (H_2_O_2_) and monitored miR-500a-5p expression thereafter. Our results showed that non-lethal concentrations of H_2_O_2_ in both cell lines induced miR-500a-5p overexpression of more than two-fold (Fig. 3a). Of note, we used lower H_2_O_2_ concentrations in MCF-7 cells due to higher toxicity in this cell line (Fig. 3a and data not shown). Interestingly, the passenger strand miR-500a-3p was also overexpressed, although to a lower degree, in response to H_2_O_2_ treatment (Fig. 3a). Moreover, we also studied the expression of two of the oxidative stress targets of miR-500a-5p identified above, *TXNRD1* and *NFE2L2*. In both cases, miR-500a-5p induction in response to H_2_O_2_ was associated with downregulation in both cell lines, although this was only significant in T47D cells (Fig. 3b).

These data suggest that miR-500a-5p does not only target oxidative stress response genes, but it is itself modulated under oxidative stress conditions.

### miR-500a-5p target expression is associated with ER+ breast cancer survival

**Figure 4:**
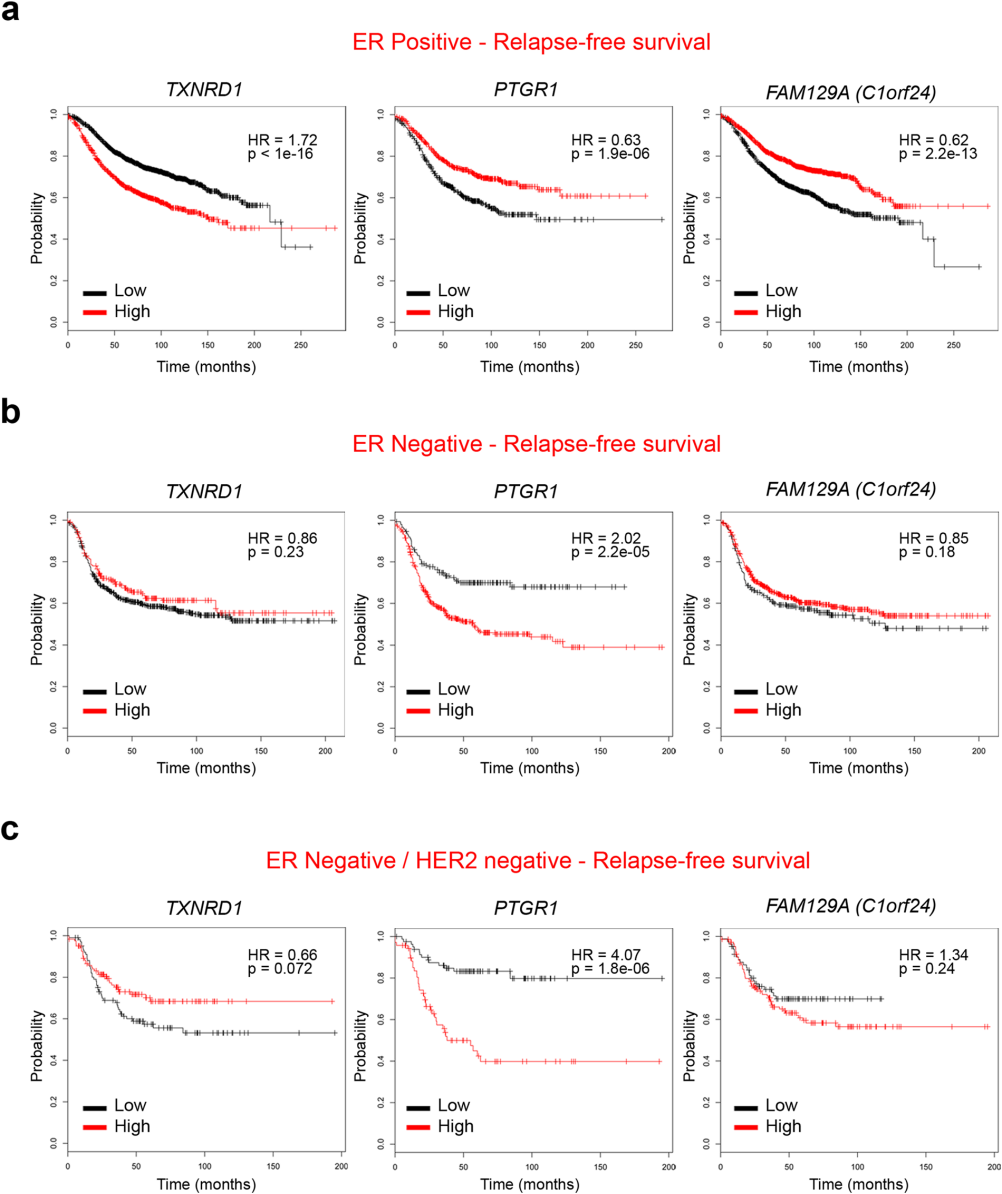
Survival analysis with identified miR-500a-5p targets (KM Plotter dataset). The breast cancer dataset from Kaplan Meier Plotter (Gyorffy B. *et al*.) was used to test for survival prediction capacity of miR-500a-5p oxidative stress targets (Table 1) in ER+ (**a**) ER− (**b**), and ER−/HER2− (**c**) breast cancer samples. Cox regression model was used for each gene to predict relapse-free survival. The three most significant associations (lowest p values) are shown (remaining four associations are shown in Fig. S1). Samples are divided into Low (black) and High (red) expression groups for each gene. Hazard ratio (HR) and P value for each association are shown within each plot.

We examined associations between miR-500a-5p targets discovered above and ER+ breast cancer survival using multiple published datasets: METABRIC (n = 1852, 77% ER+)^18^ and combination of datasets provided by KM plotter^19^ (see Methods) (n = 2862, 72% ER+). We focused our analyses on the 16 miR-500a target genes with at least two fold-change in expression after miR-500a-5p modulation (Table 1). Consistent with previous findings of positive association between mortality and miR-500a activity^7^, many of miR-500 targets displayed inverse association with breast cancer mortality (i.e. hazard ratios (HRs) below one) (Table 1). Six miR-500 target genes were consistently associated with mortality in the two datasets (i.e. *AKR1C4*, *AKR1C2*, *TXNRD1*, *PTGR1*, *HMOX1* and *FAM129A*) (Figs 4a and S4a). However, two of them, the oxidative stress genes *TXNRD1 and HMOX1*, were associated with increased mortality (HRs > 1). This is contrary to expectations for a direct miR-500a target, and suggests that dissociation between miR-500a-5p activity and degradation of some of its targets may be involved in breast cancer progression. Of note, this analysis also shows that the predictive power of several miR-500 targets is restricted to ER+ breast cancer (no significant association in ER− breast cancer subjects) (Figs 4b, S4 and S5), as expected from the reported specific association between miR-500a activity and ER+ breast cancer.

Large scale unbiased methods of miRNA target identification are a well-known strategy to uncover biological networks. In the present study, we performed a systematic identification of miR-500a targets. This miRNA is located in chromosome X, which is known to be heavily enriched in miRNA sequences (about 7% of miRNAs, or 113 miRNAs in total)^20^. Mir-500a is close to an estrogen receptor binding site, and within an intron of the chloride voltage-gated channel 5 (*CLCN5*) gene. It is clustered (within 10 kb) with hsa-mir-532, hsa-mir-188, hsa-mir-362, hsa-mir-501, hsa-mir-500b, hsa-mir-660, and hsa-mir-502 (miRDB). In addition to estrogen receptor, the expression of this whole cluster can be under the control of IL4^21^ which is a cytokine linked to breast cancer metastasis^22^. Currently, only three genes which are negative regulators of NF-kB signaling (namely *CYLD*, *TAX1BP1* and *OTUD7B*) have been discovered as validated targets for miR-500a in gastric cancer^8^. Of these, only *TAX1BP1* was common to our target list (Table S1), a finding potentially explained by differences in cellular context. Overall, we show that miR-500a-5p controls the expression of multiple oxidative stress genes, and is itself overexpressed under oxidative stress conditions. Such targets include *NFE2L2*, a gene whose downregulation has been associated with poor outcome in breast cancer patients^23^. Among differentially oxidative stress targets we found Prostaglandin Reductase 1 (*PTGR1*) and Thioredoxin Reductase 1 (*TXNRD1)*, one target predicted by *in silico* tools. Expression of these and five additional miR-500a targets identified here was able to independently predict overall breast cancer survival. This capacity of risk prediction was limited to ER+ breast cancers in five out of seven targets, in line with the described association between miR-500a activity and this subtype of breast cancers^7^.

Differential response to oxidation may represent a key factor in breast cancer prognosis. Alternatively, differences in oxidative stress could be the result of intrinsic characteristics of more aggressive tumors. In either case, our validated list of targets provides an insight into the potential role of miR-500a-5p in breast cancer survival.

## Methods

### Cell lines, cell culture conditions, and transfections

MCF-7, T47D, ZR751 cells (ER+ breast cancer cell lines), BT474 cells (ER+/HER2+ breast cancer cell lines) and hMEC and MCF10A cells (ER−, immortalized breast epithelial cells) were grown in standard (10% fetal calf serum, 1% penicillin/streptomycin, 1% sodium pyruvate and 1% glutamine) medium. Slightly attached cells from semi-confluent culture dishes were centrifuged and plated in mammosphere conditions, as previously described^24^. Refractive spheres of at least 10 cells (total diameter of approximately 50 μm) were considered as mammospheres. Alternatively, cells were counted and tested for viability with Trypan blue after trypsinization, and plated under non-attachment conditions with mammosphere medium.

In order to simulate oxidative stress conditions, we added H_2_O_2_ (Sigma Aldrich) to the media during one week. H_2_O_2_ 100 mM was freshly prepared in PBS and then added to the media at final concentrations ranging from 0 to 100 uM.

hsa-miR-500a-5p sequence (miRCURY LNA™ microRNA mimic, Exiqon) and knock-down sequence (miRCURY LNA™ microRNA inhibitor) locked nucleic acid (LNA) (Exiqon) were used for overexpression and inhibition of miR-500a-5p, respectively. miRCURY LNA™ microRNA control (Exiqon) was used as negative (mock) control.

### qRT-PCR

Extraction of total RNA, reverse transcription to cDNA and quantitative PCR (qPCR) were performed as previously described^25^. The miRNA fraction was collected separately from the total RNA. *GAPDH* was used as housekeeping gene in qPCR assays for gene expression, while *SNORD44* was used as housekeeping miRNA.

### Expression bead arrays

Total RNA was extracted using TRIzol (Sigma) according to the manufacturer’s instructions. For transcriptome analyses, total RNA from cells transfected with Pre-miR-500a-5p, miR-500a-5p inhibitor, or miRCURY LNA^TM^ microRNA control (Exiqon) was reverse-transcribed (Ambion Illumina Total Prep) and hybridized on HT12 Human bead chips (Illumina), as previously described^9^.

Raw expression data was imported and processed using R/Bioconductor packages^26^. Data quality was inspected using boxplots for the distribution of expression signals, and inter-sample relationship using multidimensional scaling plots and unsupervised clustering. To define differentially expressed (DE) genes, we modelled experimental condition (i.e. control, miR-500a-5p overexpression or inhibition) and cell type (i.e. MCF-7 or T47D) as categorical variables in a linear regression using an empirical Bayesian approach^27^. Comparisons with an FDR-adjusted P value below 0.05 were considered statistically significant. DE genes were further analyzed to determine functional pathways and ontology enrichment using EnrichR^28,29^ and DAVID^30^ web tools. miR-500a *in silico* predictions were obtained with the online database miRDB^10^. MiRonTOP^13^ was used for predictions of miRNA enrichment on the gene expression dataset (*in silico* validation) using the following parameters of significance: AVEEXP > 4, abs(LOGFC) > 0.15, and B > 0. All expression data have been deposited to the Gene Expression Omnibus repository (GEO accession number GSE92564).

### Statistical analyses

R/Bioconductor packages were used for bead array analysis, as described above. For survival analyses the METABRIC dataset^18^ was downloaded from cBioPortal (www.cbioportal.org). Survival analysis was performed using the R package *survival*. For each gene, Cox survival model was built, using coxph() function for an interval of cutoff thresholds (from second to eighth decile) and the threshold with the lowest p-value was used (approach similar to the one used in^31^). Hazards ratio of high-vs-low expression and p-value were further used as main characteristics of the correlation of a given gene with survival. In addition, survival analysis was performed using the breast cancer dataset hosted at the KM plotter website^19^ (kmplot.com/analysis). This dataset contains expression data for 54,675 genes and survival information for 5143 breast cancer cases. For other comparisons, means and differences of the means with 95% confidence intervals were obtained using GraphPad Prism (GraphPad Software Inc.). Two-tailed student t test was used for unpaired analysis comparing average expression between classes. P values < 0.05 were considered statistically significant. Error bars in the graphs represent the standard deviation.

## Electronic supplementary material

Supplementary Figures

Supplementary Datasets
