# Supplementary material for: miR-500a-5p regulates oxidative stress response genes in breast cancer and predicts cancer survival
Source: Sci Rep. 2017 Nov 21;7:15966. doi: 10.1038/s41598-017-16226-3 (PMC5698490; doi:10.1038/s41598-017-16226-3)
Supplement: Supplementary file 1 — Supplementary Figures [file 41598_2017_16226_MOESM1_ESM.pdf]

miR-500a-5p regulates oxidative stress response genes in breast cancer and predicts cancer survival

Davide Degli Esposti [1], Vasily N. Aushev [2], Eunjee Lee [3], Marie-Pierre Cros [1], Jun Zhu [3], Zdenko Herceg [1], Jia Chen [4], Hector Hernandez-Vargas [1]

1. Epigenetics Group. International Agency for Research on Cancer (IARC), 150 Cours Albert-Thomas, 69008 Lyon, France.
2. Department of Environmental Medicine and Public Health, Icahn School of Medicine at Mount Sinai, New York, NY 10029, United States of America.
3. Department of Genetics and Genomic Sciences, Icahn Institute of Genomics and Multiscale Biology. New York, NY 10029, United States of America.
4. Department of Environmental Medicine and Public Health, Icahn School of Medicine at Mount Sinai, New York, NY 10029, United States of America. Department of Pediatrics, Icahn School of Medicine at Mount Sinai, New York, NY 10029, United States of America. Department of Medicine, Hematology and Medical Oncology, Icahn School of Medicine at Mount Sinai, New York, NY 10029, United States of America. Department of Oncological Sciences, Icahn School of Medicine at Mount Sinai, New York, NY 10029, United States of America.

Supplementary Figures

Figure S1. miR-500a-5p targets in two breast cancer cell lines.

Mir-500a-5p expression was modulated in MCF-7 and T47D cells by transfection of mimic and inhibitor. **a.** multidimensional scaling (MDS) of the normalized gene expression data was performed separately for MCF-7 (left panel) and T47D cells (right panel). Compare with the combined MDS plot shown in Figure 2b. **b.** 413 differentially expressed genes (DEGs) were obtained by comparing mimics vs. inhibitor in a linear regression model, and used for unsupervised clustering (left panel) and MDS plot (right panel). **c.** The same panel of 413 probes was used again for MDS plots separately for each cell line.

Figure S2. Target prediction with different algorithms.

a. Similar to Fig 2f, we searched for the overlap between our list of experimental targets ("Targets") and the targets predicted by miRDB ("miRDB"). In addition, we used the miRecords web tool which combines several target prediction algorithms [32]. For miR500a-5p we found a highly variable number of targets: RNAhybrid = 32724, PITA = 7251, miRanda = 3836, and miRtarget2 = 526. Of these, only 306 are common (corresponding to 236 annotated genes). The Venn diagram shows the overlap between these 236 genes ("miRecords") with "miRDB" and our identified "Targets". The overlap between our target list and miRecords is highly significant (7.6 times more than expected by chance, hypergeometric test p value < 1.095x10-19) using a conservative estimate of annotated human genes (20K). **b.** we tested for miRNA enrichment in our gene expression dataset, using the webtool MiRonTOP [13]. The resulting plots compare all annotated human miRNAs and their relationship with the full expression dataset of our in vitro experiment. The x-axis represents the enrichment (fold change) in putative targets for a given miRNA relative to the DEGs list, while the y-axis indicates the significance of that relationship. Negative relationships (gene downregulation after miRNA overexpression) are shown in green, while positive relationships are shown in red. For this particular analysis, we used the complementarity of the seed sequence to define a gene as a miRNA target, either in the UTRs (left panel) or the coding sequence (right panel). The position of miR500a-5p is indicated in both panels.

Figure S3. Functional pathway analyses.

We used the EnrichR [28,29] **(a)** and DAVID [30] **(b)** pathway/ontology tools to infer the functional role of the 369 identified targets of miR500a-5p (see Methods). Only the top categories are shown, ranked by their statistical significance.

Figure S4. Survival analysis with identified miR-500a-5p targets (METABRIC dataset).

The breast cancer dataset METABRIC was used to test for survival prediction capacity of miR-500a-5p oxidative stress targets (Table 1) in ER+ **(a)** ER- **(b)**, and ER-/HER2- **(c)** breast cancer samples. Cox regression model was used for each gene to predict relapse-free survival. The three most significant associations (lowest p values) are shown. Samples are divided into Low (black) and High (red) expression groups for each gene. Hazard ratio (HR) and P value for each association are shown within each plot.

Figure S5. Survival analysis with miR-500a-5p targets (KM Plotter dataset).

The breast cancer dataset from Kaplan Meier Plotter (Gyorffy B. et al) was used to test for survival prediction capacity of miR-500a-5p oxidative stress targets (Table 1) in ER+ **(a)** ER- **(b)**, and ER-/HER2- **(c)** breast cancer samples. Cox regression model was used for each gene to predict relapse-free survival. Samples are divided into Low (black) and High (red) expression groups for each gene. Hazard ratio (HR) and P value for each association are shown within each plot.

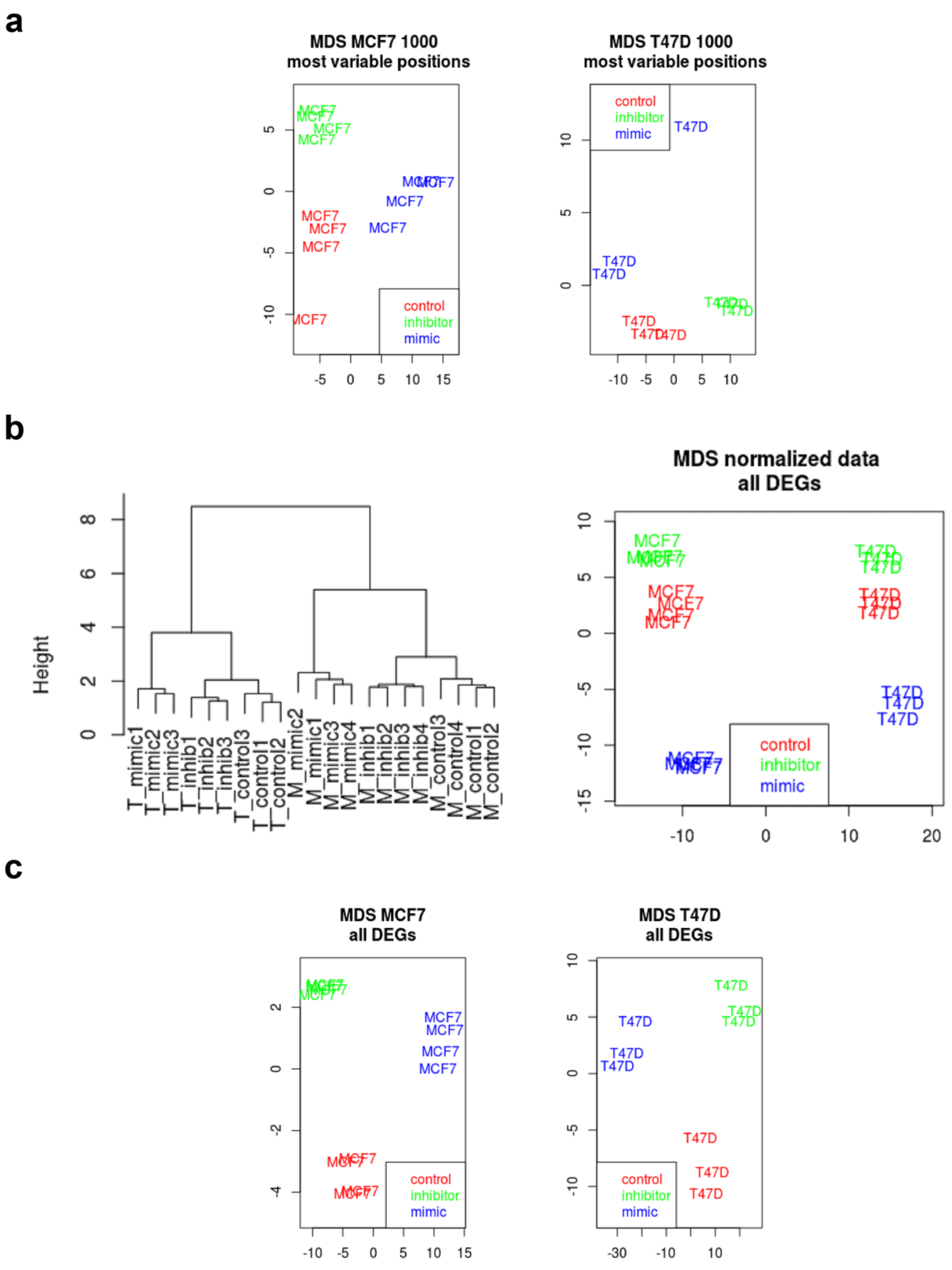
a
MDS MCF7 1000
most variable positions
MCF7
control
inhibitor
mimic
-5 0 5 10 15
-10 -5 0 5
MDS T47D 1000
most variable positions
T47D
control
inhibitor
mimic
-10 -5 0 5 10
0 5 10
b
Height
0 2 4 6 8
T_mimic1
T_mimic2
T_mimic3
T_inhib1
T_inhib2
T_inhib3
T_control3
T_control1
T_control2
M_mimic2
M_mimic1
M_mimic3
M_mimic4
M_inhib1
M_inhib2
M_inhib3
M_inhib4
M_control3
M_control4
M_control1
M_control2
MDS normalized data
all DEGs
MCF7
T47D
control
inhibitor
mimic
-10 0 10 20
-15 -10 -5 0 5 10
c
MDS MCF7
all DEGs
MCF7
control
inhibitor
mimic
-10 -5 0 5 10 15
-4 -2 0 2
MDS T47D
all DEGs
T47D
control
inhibitor
mimic
-30 -10 10
-10 -5 0 5 10

Figure S1

a

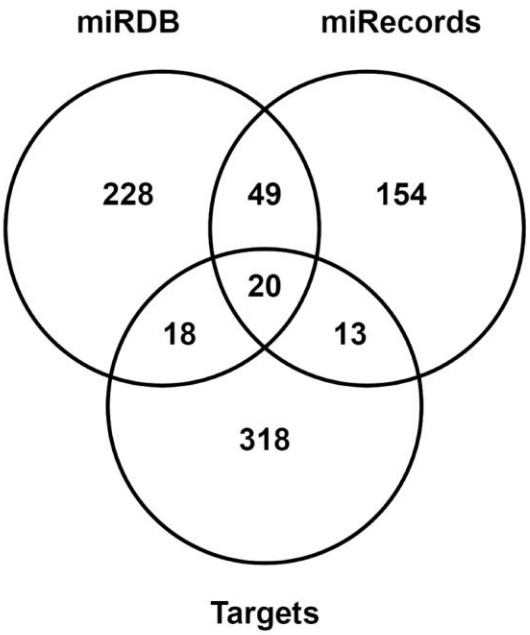
miRDB
miRecords
228
49
154
20
18
13
318
Targets

b

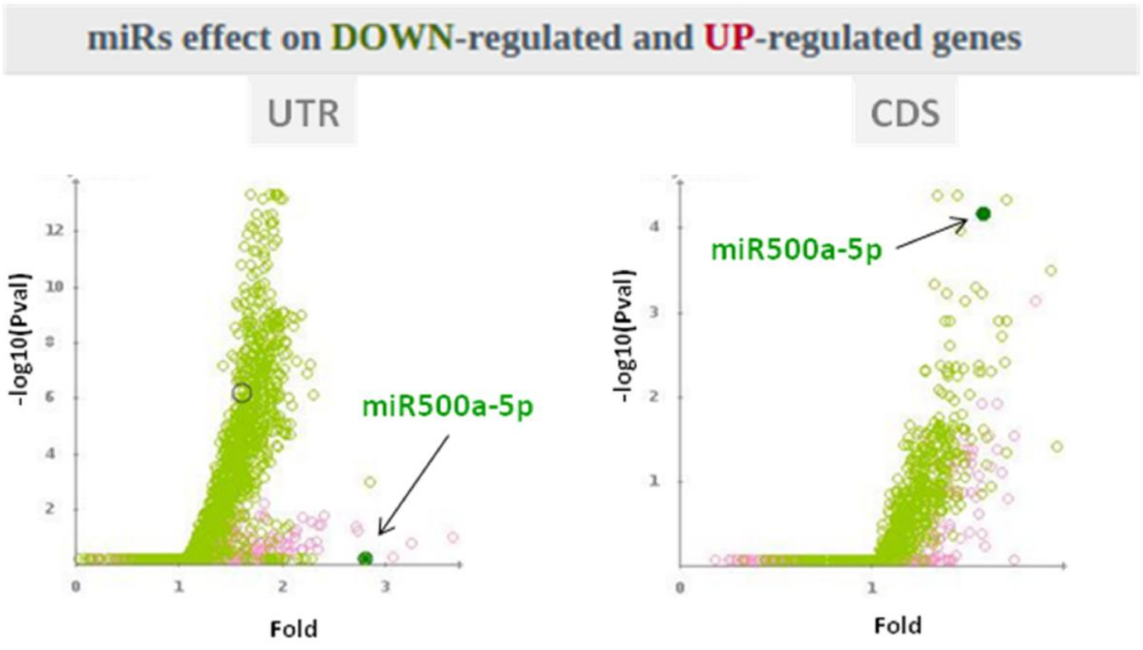
miRs effect on DOWN-regulated and UP-regulated genes
UTR
CDS
-log10(Pval)
miR500a-5p
Fold
-log10(Pval)
miR500a-5p
Fold

Figure S2

a

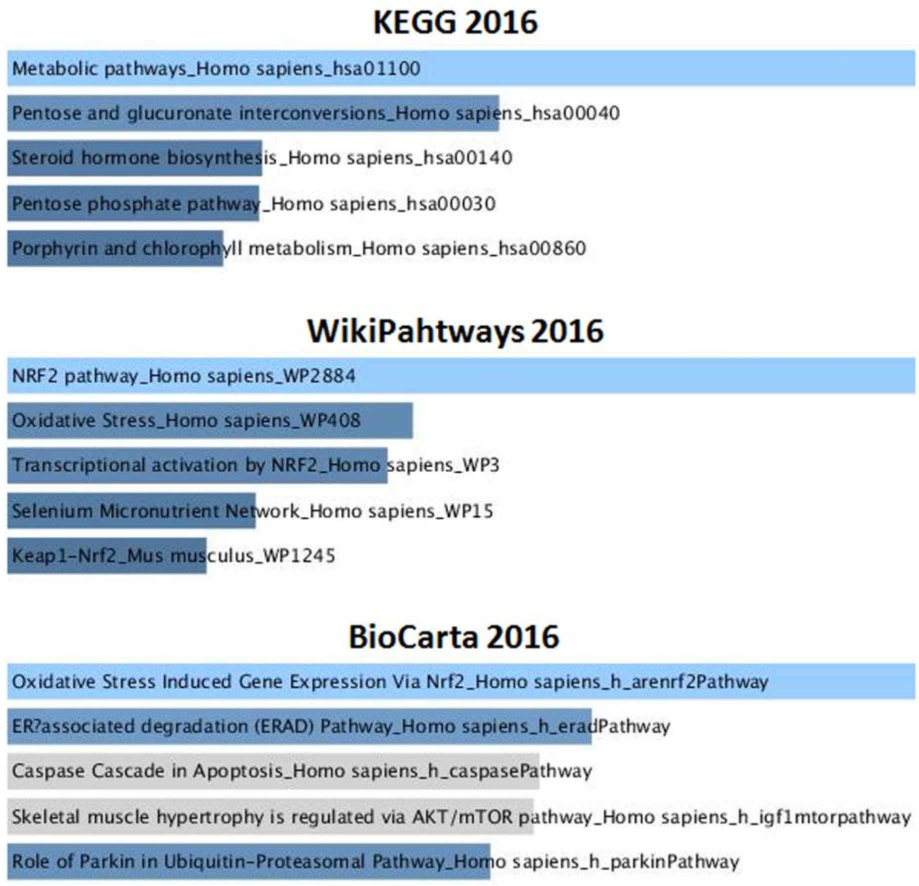

b

Functional Annotation Clustering

Enrichment Score: 7.02	G		Count	P_Value	Benjamini
oxidation-reduction process	RT		36	7.5E-12	1.1E-8
Oxidoreductase	RT		30	1.8E-9	2.6E-7
NADP	RT		17	5.4E-9	5.3E-7
nucleotide phosphate-binding region:NADP	RT		12	5.7E-9	4.5E-6
binding site:NADP	RT		7	2.4E-6	9.5E-4
oxidoreductase activity	RT		13	6.5E-5	3.2E-2
binding site:Substrate	RT		13	1.1E-3	1.9E-1

Figure S3

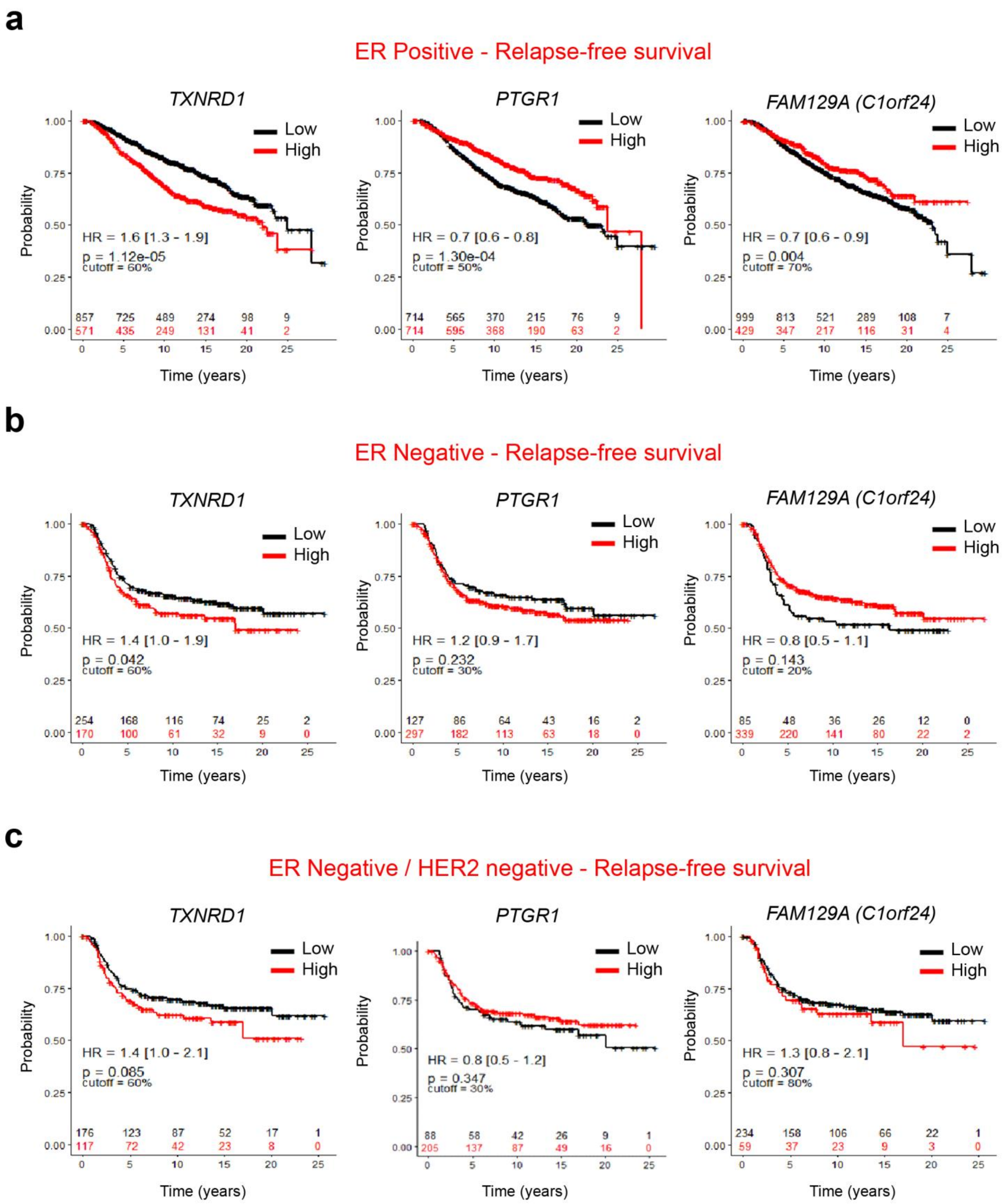
a
ER Positive - Relapse-free survival
TXNRD1
Low
High
Probability
HR = 1.6 [1.3 - 1.9]
p = 1.12e-05
cutoff = 60%
857 725 489 274 98 9
571 435 249 131 41 2
Time (years)
PTGR1
HR = 0.7 [0.6 - 0.8]
p = 1.30e-04
cutoff = 50%
714 565 370 215 76 9
714 595 368 190 63 2
FAM129A (C1orf24)
HR = 0.7 [0.6 - 0.9]
p = 0.004
cutoff = 70%
999 813 521 289 108 7
429 347 217 116 31 4
b
ER Negative - Relapse-free survival
TXNRD1
HR = 1.4 [1.0 - 1.9]
p = 0.042
cutoff = 60%
254 168 116 74 25 2
170 100 61 32 9 0
PTGR1
HR = 1.2 [0.9 - 1.7]
p = 0.232
cutoff = 30%
127 86 64 43 16 2
297 182 113 63 18 0
FAM129A (C1orf24)
HR = 0.8 [0.5 - 1.1]
p = 0.143
cutoff = 20%
85 48 36 26 12 0
339 220 141 80 22 2
c
ER Negative / HER2 negative - Relapse-free survival
TXNRD1
HR = 1.4 [1.0 - 2.1]
p = 0.085
cutoff = 60%
176 123 87 52 17 1
117 72 42 23 8 0
PTGR1
HR = 0.8 [0.5 - 1.2]
p = 0.347
cutoff = 30%
88 58 42 26 9 1
205 137 87 49 16 0
FAM129A (C1orf24)
HR = 1.3 [0.8 - 2.1]
p = 0.307
cutoff = 80%
234 158 106 66 22 1
59 37 23 9 3 0

Figure S4

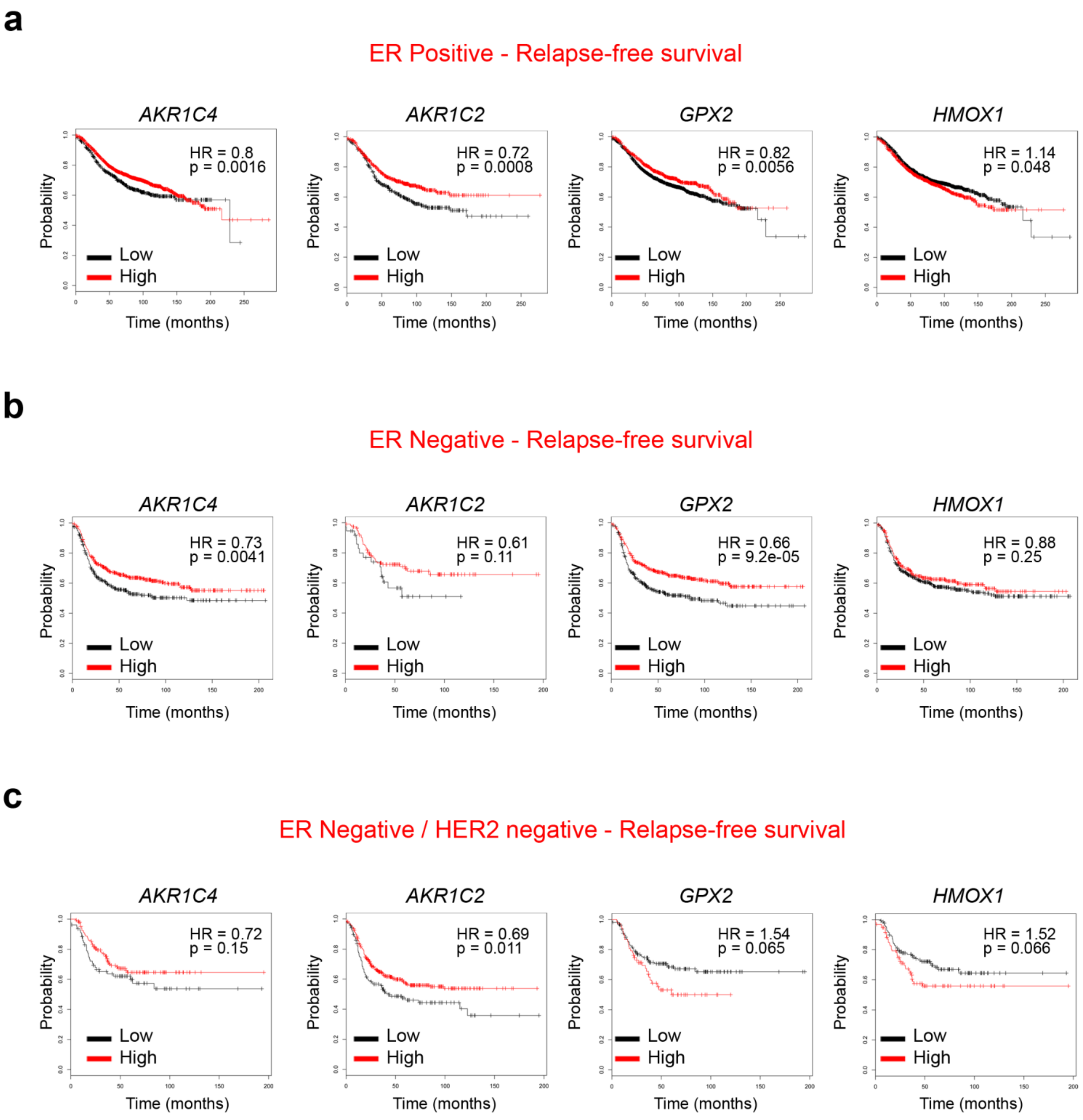
a
ER Positive - Relapse-free survival
AKR1C4
HR = 0.8
p = 0.0016
AKR1C2
HR = 0.72
p = 0.0008
GPX2
HR = 0.82
p = 0.0056
HMOX1
HR = 1.14
p = 0.048
b
ER Negative - Relapse-free survival
AKR1C4
HR = 0.73
p = 0.0041
AKR1C2
HR = 0.61
p = 0.11
GPX2
HR = 0.66
p = 9.2e-05
HMOX1
HR = 0.88
p = 0.25
c
ER Negative / HER2 negative - Relapse-free survival
AKR1C4
HR = 0.72
p = 0.15
AKR1C2
HR = 0.69
p = 0.011
GPX2
HR = 1.54
p = 0.065
HMOX1
HR = 1.52
p = 0.066
Probability
Time (months)
Low
High

Figure S5
